# Combinatory Biomarker Use of Cortical Thickness, MUNIX, and ALSFRS-R at Baseline and in Longitudinal Courses of Individual Patients With Amyotrophic Lateral Sclerosis

**DOI:** 10.3389/fneur.2018.00614

**Published:** 2018-07-30

**Authors:** Anna M. Wirth, Andrei Khomenko, Dobri Baldaranov, Ines Kobor, Ohnmar Hsam, Thomas Grimm, Siw Johannesen, Tim-Henrik Bruun, Wilhelm Schulte-Mattler, Mark W. Greenlee, Ulrich Bogdahn

**Affiliations:** ^1^Department of Neurology, University Hospital of Regensburg, Regensburg, Germany; ^2^Department of Experimental Psychology, University of Regensburg, Regensburg, Germany

**Keywords:** amyotrophic lateral sclerosis, magnetic resonance imaging, cortical thickness, MUNIX, ALSFRS-R

## Abstract

**Objective:** Amyotrophic lateral sclerosis (ALS) is a progressive neurodegenerative process affecting upper and lower motor neurons as well as non-motor systems. In this study, precentral and postcentral cortical thinning detected by structural magnetic resonance imaging (MRI) were combined with clinical (ALS-specific functional rating scale revised, ALSFRS-R) and neurophysiological (motor unit number index, MUNIX) biomarkers in both cross-sectional and longitudinal analyses.

**Methods:** The unicenter sample included 20 limb-onset classical ALS patients compared to 30 age-related healthy controls. ALS patients were treated with standard Riluzole and additional long-term G-CSF (Filgrastim) on a named patient basis after written informed consent. Combinatory biomarker use included cortical thickness of atlas-based dorsal and ventral subdivisions of the precentral and postcentral cortex, ALSFRS-R, and MUNIX for the musculus abductor digiti minimi (ADM) bilaterally. Individual cross-sectional analysis investigated individual cortical thinning in ALS patients compared to age-related healthy controls in the context of state of disease at initial MRI scan. Beyond correlation analysis of biomarkers at cross-sectional group level (*n* = 20), longitudinal monitoring in a subset of slow progressive ALS patients (*n* = 4) explored within-subject temporal dynamics of repeatedly assessed biomarkers in time courses over at least 18 months.

**Results:** Cross-sectional analysis demonstrated individually variable states of cortical thinning, which was most pronounced in the ventral section of the precentral cortex. Correlations of ALSFRS-R with cortical thickness and MUNIX were detected. Individual longitudinal biomarker monitoring in four slow progressive ALS patients revealed evident differences in individual disease courses and temporal dynamics of the biomarkers.

**Conclusion:** A combinatory use of structural MRI, neurophysiological and clinical biomarkers allows for an appropriate and detailed assessment of clinical state and course of disease of ALS.

## Introduction

Amyotrophic lateral sclerosis (ALS) is a rapidly progressive neurodegenerative disorder affecting upper and lower motor neurons as well as non-motor systems (1). The degeneration of motor neurons results in muscular fasciculation, progressive weakness, and eventual paralysis (2). Average survival in ALS is 3–5 years, but patients evidently vary in phenotype and disease progression (2, 3). The great clinical heterogeneity in ALS is reflected by different phenotypes with variability regarding the involvement of upper motor neuron (UMN) and lower motor neuron (LMN) signs, site of onset (bulbar, limb), rate of progression, and involvement of neurobehavioral deficits (2, 4). Therefore, clinical and biological biomarkers are helpful in describing disease severity and progression (3).

Magnetic resonance imaging (MRI) has produced potential biomarkers that clarify the role of brain structure and function in the progress of the disease (5, 6). In structural morphometric studies, cortical thickness compared to surface and volume was most sensitive to disease-related changes (7). A variety of studies investigating structural surface-based morphometry showed reduced cortical thickness primarily in the precentral cortex (8–16). Cortical thinning was not restricted to the primary motor cortex. Several studies reported cortical thinning to spread to non-motor cortex areas like the temporal, frontal, parietal, and postcentral cortex (8, 10, 15). However, not all published MRI studies detected alterations in the cortical thickness (17) or cortical volume (18, 19) of the precentral cortex of ALS patients. Essentially, precentral cortical thinning was reported to be focal, and dependent on the clinical phenotype, rate of progression, and age (8, 11, 13). Additionally, several longitudinal MRI studies revealed no further cortical thinning of the precentral cortex in the course of disease (9, 14–17).

In addition to MRI, clinical and electrophysiological biomarkers are among the most currently used and prominent biomarkers (20). The widely used ALS-specific functional rating scale revised (ALSFRS-R) and its subscales are correlated with survival (3). However, correlations between precentral cortical thickness and ALSFRS-R scores were rather weak (10, 21) or not detected in several neuroimaging studies so far (9, 12, 13, 16, 22). While MRI is considered a suitable biomarker for UMN function, neurophysiological motor unit number estimation (MUNE) and motor unit number index (MUNIX) are treated as biomarkers for the estimation of functional lower motor units (23, 24). MUNE is calculated from the division of maximal compound muscle action potential (CMAP) by the mean surface single motor unit action potential (SMUP) (25). In contrast, MUNIX is derived from a mathematical model based on CMAP and electromyographic surface interference patterns (SIP) (26). MUNE and MUNIX scores are inter-correlated in ALS patients (23). As the acquisition of MUNIX is easier and less time consuming than that of MUNE, MUNIX has become a promising biomarker of motor unit loss (27, 28). MUNIX scores were correlated with ALSFRS-R scores (26), but they declined faster than ALSFRS-R scores over time in ALS patients (29). Only few studies investigated the relationship between neurophysiological biomarkers and cortical thickness and failed to find a significant correlation with MUNE or other motor evoked potential indices (21, 30). To our knowledge, no published study investigated correlations between cortical thickness and MUNIX as a biomarker potentially affected by both lower and UMN function (23).

Aim of the study was to investigate individual states of cortical thinning of the precentral and postcentral cortex in a limb-onset ALS sample with respect to young-onset, and slow disease progression. It is the first study to analyze combinatory biomarker use of MRI cortical thickness, neurophysiological MUNIX, and routine ALSFRS-R in both cross-sectional group analysis of the whole sample, and in longitudinal monitoring exploring differences in temporal dynamics between biomarkers in a subgroup of slow progressive ALS patients.

## Materials and methods

### Participants

Cross-sectional group analysis included 20 limb-onset classical ALS patients (5 females, *M* = 48 years, *SD* = 11) compared to 30 age-related healthy controls (14 females, *M* = 45 years, *SD* = 13). Mean age of ALS patients was lower than that reported in other ALS studies, as the sample included several young-onset patients. Mean ALSFRS-R score across all 20 patients at the time point of first MRI scan was 36 score points (*SD* = 8; range: 23–48). The sample included both slow and fast progressive ALS patients indicated by disease progression rates (*M* = 0.51, *SD* = 0.27; range: 0.00–1.00). The presence of both UMN and LMN signs in all patients allowed no clear differentiation in UMN or LMN predominance of disease. Patients' characteristics are summarized in Table 1. These included age (in ranges of years), ALSFRS-R sum scores and neurophysiological MUNIX scores for left and right ADM upon initial MRI scan, time interval between symptom onset and initial T1 MRI scan (in months), onset of disease (arm, leg), and progression rates [(48-ALSFRS-R)/months since symptom onset] (8). Genetic background of ALS was exhibited in one patient only (patient 8). All other patients were diagnosed as sporadic ALS.

**Table 1:** Patients' characteristics at baseline.

**#**	**Range of age**	**ALSFRS-R [0 48]**	**MUNIX left**	**MUNIX right**	**Time span since onset**	**Onset**	**Progression rate**
1	21–25	30	6.63	10.43	49	Arm	0.37
2	26–30	35	8.20	29.21	28	Arm	0.46
3	31–35	24	6.94	2.83	28	Leg	0.86
4	41–45	24	*	*	28	Leg	0.86
5	41–45	41	54.12	38.00	19	Leg	0.37
6	41–45	28	4.85	0.70	38	Arm	0.53
7	41–45	46	*	*	16	Arm	0.13
8	46–50	39	138.40	41.88	56	Leg	0.16
9	46–50	48	170.10	187.30	19	Leg	0.00
10	46–50	24	48.37	18.51	24	Arm	1.00
11	46–50	46	213.60	193.00	3	Leg	0.67
12	46–50	40	114.50	97.18	21	Leg	0.38
13	46–50	38	*	*	25	Leg	0.40
14	51–55	42	27.25	21.62	7	Arm	0.86
15	51–55	35	*	*	33	Leg	0.39
16	56–60	38	123.90	101.60	29	Leg	0.34
17	56–60	44	126.90	0.00	13	Leg	0.31
18	61–65	42	83.10	119.00	10	Leg	0.60
19	61–65	24	12.34	9.66	33	Leg	0.73
20	66–70	21	6.94	2.83	36	Leg	0.75

Summary of baseline characteristics of all 20 limb-onset ALS patients including age (in ranges of years), ALSFRS-R sum score upon initial MRI scan (48 in clinical non affected), neurophysiological MUNIX scores for left and right ADM upon initial MRI scan, the length of time span between symptom onset and initial MRI scan in months, onset of disease (arm, leg), and progression rate. In four out of 20 ALS patients (marked with ^*^), neurophysiological assessment was still conducted using MUNE technique (patient 4: left MUNE = 2, right MUNE = 1; patient 7: left MUNE = 3, MUNE right = 77; patient 13: left MUNE = 275, right MUNE = 85; patient 15: left MUNE = 240, right MUNE = 120). No MUNIX scores were obtained in these four patients. Progression rates were calculated by [48-ALSFRS-R sum score/months since symptom onset; see (8)]. Neuropsychological assessment using the Edinburgh Cognitive and Behavioral ALS Screen (ECAS) was conducted only in patient 3 (129/136 score points) and patient 9 (100/136 score points).

All patients received standard Riluzole treatment and additional G-CSF (granulocyte-colony stimulating factor, Filgrastim) treatment on a named patient basis. Application modes and doses of G-CSF were individually adapted, treatment duration was up to 7 years. For safety and monitoring of progression, structural MRI and MUNIX were assessed every 3 months. ALSFRS-R scores were acquired monthly, but were integrated in the analysis only at the time points of MRI scanning. MRI cortical thickness was combined with ALSFRS-R sum scores, and MUNIX scores for left and right ADM in both cross-sectional analysis and longitudinal biomarker monitoring.

The unicenter project was carried out in accordance with the Declaration of Helsinki (31) and approved by the ethics committee at the University of Regensburg (ethics approval: 15-101-0106). Written informed consent was obtained prior to participation in all participants.

### Data acquisition

Structural MRI was conducted at a 1.5 Tesla clinical scanner (Aera, Siemens Medical, Erlangen, Germany). For each patient, a high-resolution T1-weighted structural scan was obtained by a magnetization prepared rapid gradient echo sequence (MPRAGE; time-to-repeat TR: 2220 ms, time-to-echo TE: 5.97 ms, flip angle FA: 15°, voxel size: 1 × 1 × 1 mm3, field of view FOV: 256 × 256 mm^2^, 176 sagittal slices covering the whole brain).

MUNIX estimates the number of motor units in a muscle by a mathematical algorithm involving both the compound muscle action potentials (CMAP) and the continuous electromyographic surface interference pattern (SIP) of the muscles (23, 27). In contrast to original MUNIX, MUNIX recordings of this project implicated continuous SIP recordings during increasing muscle contraction. SIP data were modified by baseline correction, filter settings, rectifications, and SIP intervals. Artifacts were corrected by exclusion of SIP intervals below a specified baseline threshold. As MUNIX was introduced more recently as a neurophysiological biomarker, four out of 20 ALS patients received the assessment of MUNE only (see Table 1).

### MRI data preprocessing

T1-weighted structural images were reconstructed by Freesurfer software version 5.3 (Martinos Center for Biomedical Imaging, Charlestown, MA). The reconstruction procedure included automatic segmentation of gray matter and subcortical white matter (32) and tessellation and registration of the cortical surface to a spherical atlas (33). For group analysis, T1-weighted images of the 20 individual patients' brains were registered to the Freesurfer average structural brain by using the Freesurfer linear and non-linear image registration tools (FLIRT, FNIRT).

### ROI definition

**Figure 1:**
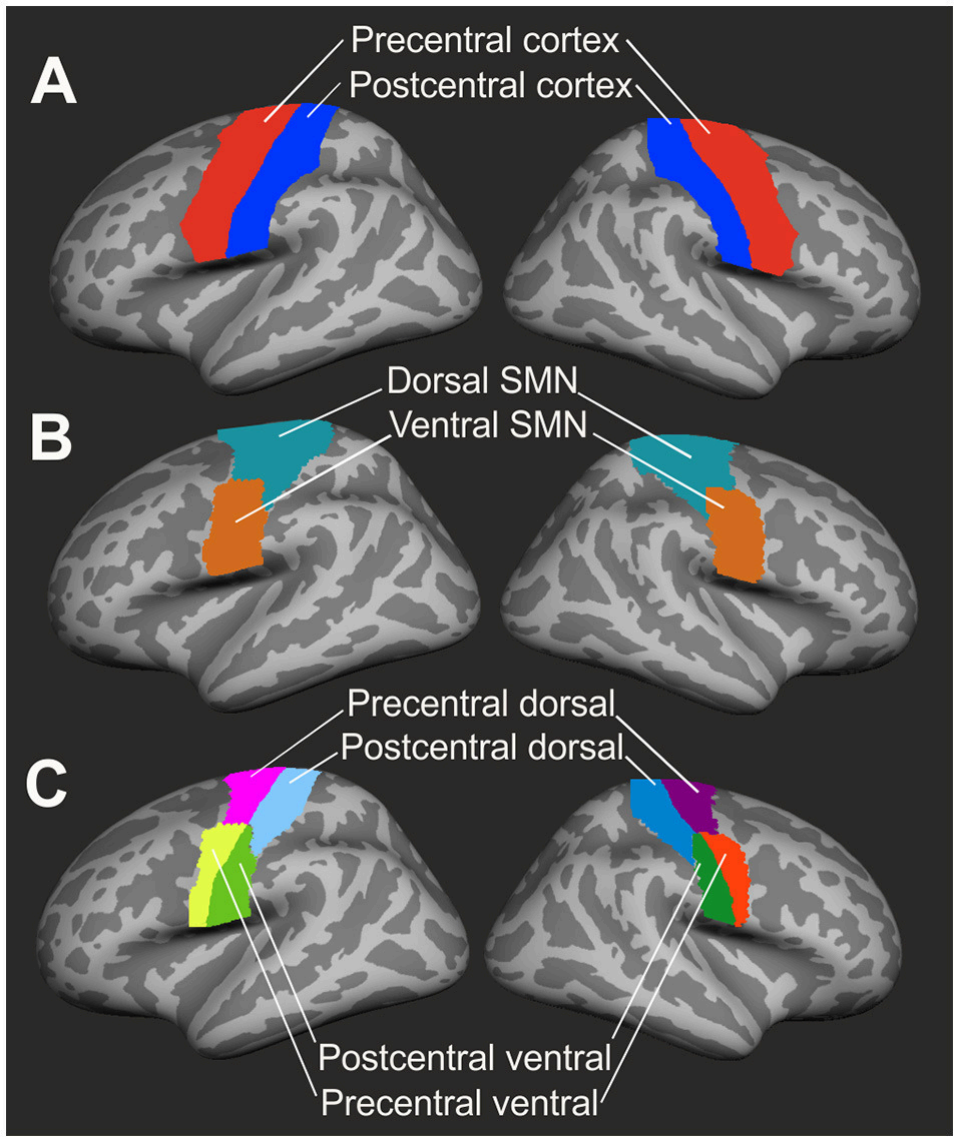
Definition of regions of interest. **(A)** Precentral (red) and postcentral (blue) cortex were identified by the Desikan-Killiany parcellation atlas (34). **(B)** The resting-state-fMRI based atlas of Yeo et al. (35) was used to define dorsal (marine blue) and ventral (brown) segments of the sensorimotor network (SMN). **(C)** Final subdivision of the precentral and postcentral cortex into dorsal and ventral segments resulted in four ROIs (precentral dorsal, precentral ventral, postcentral dorsal, postcentral ventral) in both left and right hemisphere. ROIs were mapped upon the Freesurfer average brain surface.

Cortical thickness analysis focused on precentral and postcentral regions of interest (ROI) as defined by the Desikan-Killiany parcellation atlas [(34); see Figure 1A]. Dorsal and ventral subdivisions of the sensorimotor network were defined by a resting-state-functional MRI (fMRI) data-based atlas (35) in volumetric MNI (Montreal Neurological Institute) space. These ROIs were subsequently registered to the Freesurfer volumetric space and then to the Freesurfer average brain surface (see Figure 1B). As all ROIs were mapped upon the Freesurfer average brain space, ROIs were identically sized in each patient and healthy control.

### Computation of cortical thickness

Cortical thickness was computed according to a workflow recommended by Freesurfer software. Individual surface-based cortical thickness data were mapped upon the Freesurfer average brain surface. By the use of a segmentation statistical tool of Freesurfer software, cortical thickness in each of the four ROIs (PreD: precentral dorsal, PreV: precentral ventral, PoD: postcentral dorsal, PoV: postcentral ventral, see Figure 1C) was calculated and extracted as a mean value across vertices.

### Cross-sectional group analysis

Group-analysis of mean cortical thickness was conducted using a repeated measures ANOVA with the within-subject factors region (PreD, PreV, PoD, PoV) and hemisphere (left vs. right), and the between-subject factors group (ALS vs. controls), gender (male vs. female), and the covariate age. Differences in cortical thickness between regions were investigated by paired *t*-tests and differences between patients and controls were analyzed using independent-samples *t*-tests. *T*-tests were corrected by Bonferroni correction. Correlation analyses were conducted to investigate relations between cortical thickness, ALSFRS-R sum scores and subscores, and MUNIX by the Bravais-Pearson correlation coefficient. Significance level was set to *p* < 0.05. Multiple comparison errors were controlled by Bonferroni correction procedure in *post-hoc* analyses.

### Individual cortical thickness analysis

In addition to cross-sectional group analysis, this project focused on the interindividual variability of cortical thinning. For this purpose, we compared the cortical thickness of all 20 patients to age-related controls, resulting in individual *z*-transformed deviations of cortical thickness from healthy control level. As age effects on cortical thickness are well described (36), ALS patients were compared to one out of two possible age groups. Based on the mean age of ALS patients, the 30 healthy controls were differentiated into two comparably sized subgroups (1: age < 48 years, *n* = 17; 2: age ≥48 years, *n* = 13). Furthermore, *z*-transformed deviations of cortical thickness from healthy controls as well as biomarkers MUNIX and ALSFRS-R were monitored in four individual slow progressive ALS patients over a time course of at least 18 months (patient 1, 2, 8, 9, see Table 1). All other patients exhibited MRI time courses of a maximum of 9 months only (3 scans: *n* = 2; 2 scans *n* = 6; 1 scan: *n* = 8) due to high disability and lack of T1 MRI data.

## Results

### Cross-sectional group analysis

**Figure 2:**
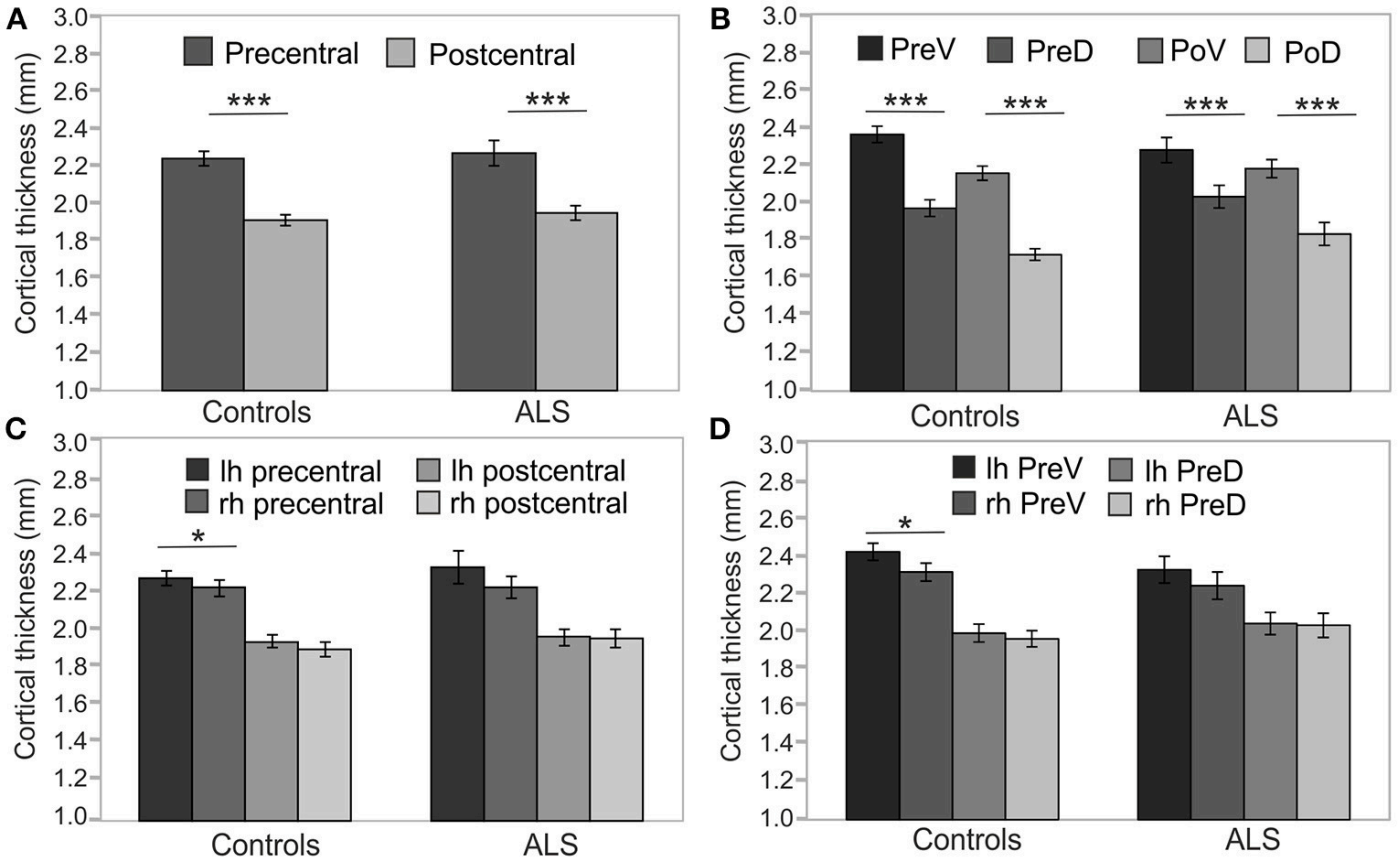
Precentral and postcentral cortical thickness. Cross-sectional analyses of cortical thickness of ALS patients (*n* = 20) and healthy controls (*n* = 30). **(A)** Precentral cortical thickness averaged across left (lh) and right (rh) hemisphere was significantly higher than postcentral cortical thickness in both ALS patients and healthy controls. **(B)** Cortical thickness of ventral segments of both precentral (PreV) and postcentral (PoV) cortex were similarly higher than in dorsal segments (PreD, PoD) in both ALS patients and healthy controls. Hemispheric differences were detected only in the precentral **(C)** and precentral ventral cortex **(D)** of healthy controls. Significance level was set to *p* < 0.05. Bonferroni correction was used for multiple comparisons. Lh, left hemisphere; Rh, right hemisphere; PreV, precentral ventral; PreD, precentral dorsal; PoV, postcentral ventral; PoD, postcentral dorsal. Asterisks refer to the height of *p*-value: ^*^p < 0.05, ^***^p < 0.001.

As Mauchly's test indicated that the assumption of sphericity was violated [χ^2^_(5)_ = 22.42, *p* < 0.001], degrees of freedom were corrected using Greenhouse-Geisser estimates of sphericity (ε = 0.79). Cortical thickness was not significantly different between patients and healthy controls [*F*_(1, 45)_ = 1.314; *p* = 0.258] at cross-sectional group level. Cortical thickness significantly varied across cerebral regions [*F*_(3, 135)_ = 23.351, *p* < 0.001] and with respect to age [*F*_(1, 45)_ = 21.776, *p* < 0.001]. Cortical thickness was significantly higher in precentral than in postcentral regions in both ALS patients [*T*_(19)_ = 8.584, *p* < 0.05, corrected], and healthy controls [*T*_(29)_ = 16.521, *p* < 0.05, corrected] (Figure 2A). Ventral subdivisions of precentral and postcentral cortex showed greater cortical thickness than dorsal subdivisions in both ALS patients [*T*_(19)_ = 9.906, *p* < 0.05, corrected] and healthy controls [*T*_(29)_ = 12.389; *p* < 0.05, corrected] (Figure 2B). The ANOVA revealed no significant main effect of hemisphere, as significant hemispheric differences in cortical thickness were restricted to the precentral [*T*_(29)_ = 3.445, *p* < 0.05, corrected] (Figure 2C) and precentral ventral cortex [*T*_(29)_ = 3.596, *p* < 0.05, corrected] of healthy controls (Figure 2D). ALSFRS-R sum scores correlated with cortical thickness of the precentral ventral cortex (*r* = 0.570, *p* = 0.009) and the postcentral ventral region (*r* = 0.481, *p* = 0.032). Cortical thickness did not significantly correlate with MUNIX scores for left and right ADM in any ROI. MUNIX scores for the left (*r* = 0.767, *p* < 0.05, corrected) and right (*r* = 0.791, *p* < 0.05, corrected) ADM correlated with ALSFRS-R sum scores. Highest correlations of cortical thickness with ALSFRS-R subscores were found for turning (PreV: *r* = 0.501, *p* = 0.024; PoV: *r* = 0.652, *p* = 0.002), walking (PoV: *r* = 0.603, *p* = 0.005), and cutting (PreV: *r* = 0.453, *p* = 0.045). MUNIX scores predominantly correlated with ALSFRS-R subscores on handwriting (left ADM: *r* = 0.637, *p* = 0.008; right ADM: *r* = 0.678, *p* = 0.005), cutting (left ADM: *r* = 0.840, *p* < 0.001; right ADM: *r* = 0.834, *p* < 0.001), dressing (left ADM: *r* = 0.793, *p* < 0.001, right ADM: *r* = 0.806, *p* < 0.001), turning (left ADM: *r* = 0.609, *p* = 0.012; right ADM: *r* = 0.663, *p* = 0.007), and climbing stairs (left ADM: *r* = 0.563, *p* = 0.023; right ADM: *r* = 0.611, *p* = 0.016). ALS patients were separated *post-hoc* in arm-onset (*n* = 7) and leg-onset (*n* = 13) groups. Arm-onset patients showed significantly lower MUNIX scores for ADM (left: *M* = 19, *SD* = 19; right: *M* = 16, *SD* = 11) than leg-onset patients (left: *M* = 95, *SD* = 69; right: *M* = 72, *SD* = 72) [left ADM: *T*_(14)_ = −3.399, *p* < 0.05, corrected; right ADM: *T*_(14)_ = −2.506; *p* = 0.029]. Arm-onset and leg-onset patients did not significantly differ in disease progression, ALSFRS-R sum scores and subscores.

### Variability of cortical thinning

Thirty healthy controls were differentiated into two groups of age (1. age < 48 years, 2. age ≥48 years, see section Individual Cortical Thickness Analysis). In each of the two subgroups, means of cortical thickness of all precentral and postcentral ROIs (see Table 2) were calculated. These mean values were used as reference values for the calculation of *z*-transformed deviations of ROI-specific cortical thickness of individual ALS patients (for patient numbers see Table 1) from healthy control level.

**Table 2:** References values of cortical thickness.

		**Lh PreV (mm)**	**Rh PreV (mm)**	**Lh PreD (mm)**	**Rh PreD (mm)**	**Lh PoV (mm)**	**Rh PoV (mm)**	**Lh PoD (mm)**	**Rh PoD (mm)**
1	*M*	2.542	2.442	2.106	2.068	2.276	2.205	1.804	1.798
	*SD*	0.205	0.139	0.194	0.158	0.165	0.188	0.158	0.163
2	*M*	2.249	2.132	1.827	1.807	2.072	2.016	1.663	1.584
	*SD*	0.184	0.275	0.242	0.232	0.198	0.244	0.141	0.145

*Cortical thickness (mm) of healthy control participants subdivided into two age groups (1: age < 48 years, 2: age ≥48 years). Mean age of the two subgroups: Group 1: M = 36, SD = 7, n = 17; Group 2: M = 52, SD = 15, n = 13. Lh, left hemisphere; Rh, right hemisphere; PreV, precentral ventral; PreD, precentral dorsal; PoV, postcentral ventral; PoD, postcentral dorsal*.

**Figure 3:**
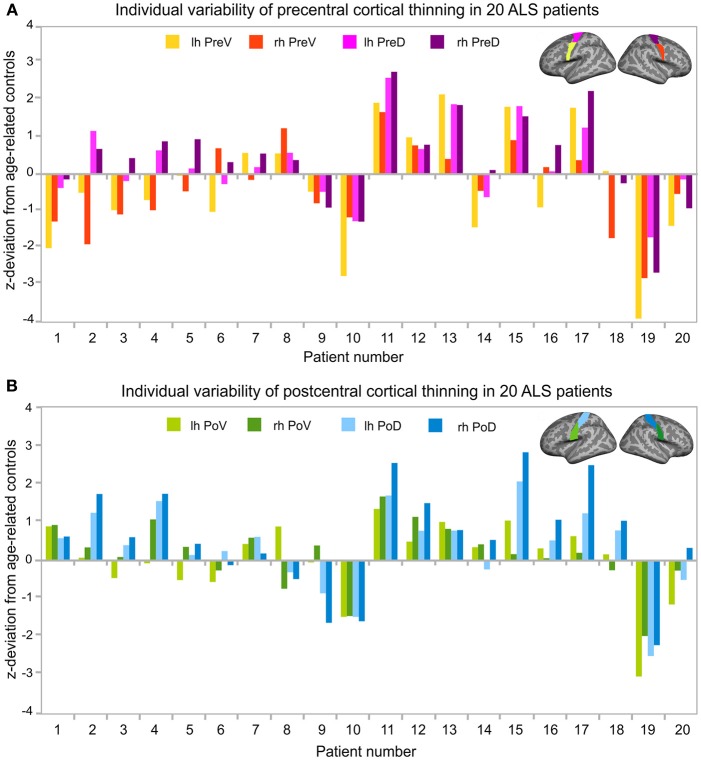
Individual variability of cortical thinning in ALS patients. Z-transformed deviation of cortical thickness from age-related healthy controls (see Table 2) in all 20 individual ALS patients. Patient numbers refer to Table 1. Patients were sorted by age. *Z*-transformed deviations of cortical thickness were considered relevant at least one deviation from healthy controls. **(A)** Individual variability of cortical thickness of the left (lh, yellow) and right (rh, red) precentral ventral (PreV) and left (lh, pink) and right (rh, purple) precentral dorsal (PreD) ROIs. **(B)** Deviations of individual cortical thickness of the left (lh, light green) and right (rh, dark green) postcentral ventral (PoV) and the left (lh, bright blue) and right (rh, dark blue) postcentral dorsal (PoD) region in the same 20 individual patients. Precentral **(A)** and postcentral **(B)** ROIs were visualized upon the Freesurfer average brain surface. Lh, left hemisphere; Rh, right hemisphere; PreV, precentral ventral; PreD, precentral dorsal; PoV, postcentral ventral; PoD, postcentral dorsal.

Cortical thickness alterations below at least one deviation from healthy control level were detected in eleven out of twenty patients *(*patients 1, 2, 3, 4, 6, 10, 14, 16, 18, 19, 20) and marginally indicated in two patients (patients 5, 9). Cortical thinning was primarily observed in the precentral cortex, especially in the ventral segment (Figure 3A). Most pronounced cortical thinning in all precentral ROIs was detected in patient 10 and patient 19. Leg-onset patient 19 was characterized by older age (range: 61–65 years), low ALSFRS-R score (24 score points), low MUNIX scores of ADM, and high disease progression rate (0.73). Patient 10 was much younger (range: 46–50 years), but showed low ALSFRS-R score (24 score points), and the highest progression rate (1.00) of the entire patient sample (see Table 1). The two youngest ALS patients (patients 1–2, age ranges: 21–25 and 26–30 years) shared similar patterns of cortical thinning, similar mode of disease (arm-onset), low ALSFRS-R scores, low MUNIX scores for ADM, and similar disease progression rates (see Table 1, see Figure 3A). Cortical thinning in the postcentral cortex was detected in four patients (patients 9, 10, 19, 20; Figure 3B). Three out of these four patients also exhibited evident precentral cortical thinning. Leg-onset patient 9 stood out of the sample with the highest ALSFRS-R score, high MUNIX scores for ADM, lowest progression rate (0.00), and more pronounced cortical thinning of the postcentral cortex than of the precentral cortex. Increased levels of cortical thickness above healthy control level were more prominent in the postcentral cortex than in the precentral cortex. Seven patients exhibited unremarkable levels of cortical thickness (patients 7, 8, 11, 12, 13, 15, 17). Six (patients 7, 8, 12, 13, 15, 17) out of these seven patients exhibited disease progression rates less or equal to 0.40 (see Table 1).

### Longitudinal monitoring of cortical thickness, ALSFRS-R, and MUNIX

**Figure 4:**
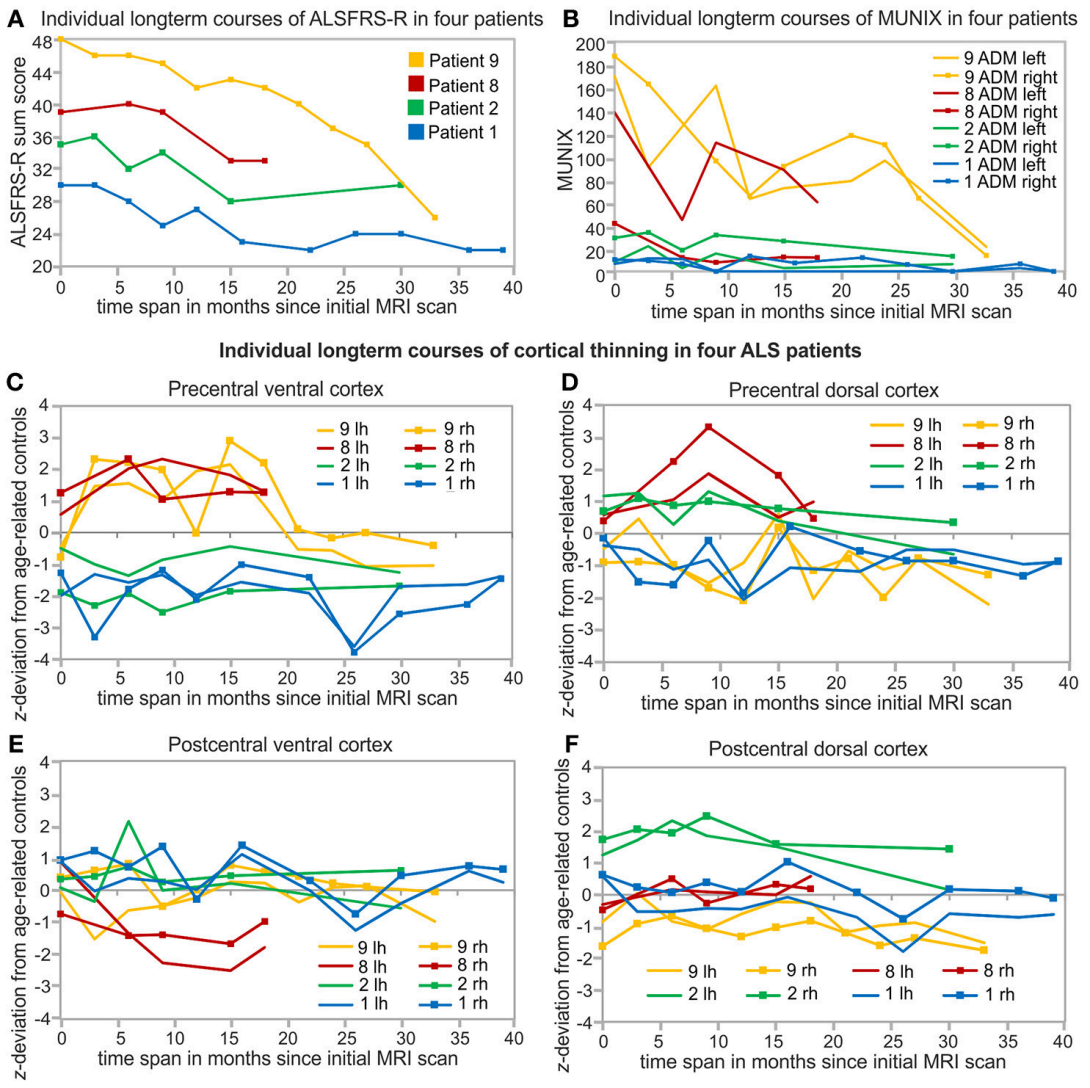
Individual and long-term biomarker monitoring over at least 18 months. Arm-onset and young-onset patients (patient 1, 2) showed higher progression rates (see Table 1) than leg-onset similarly aged patients 8 and 9. Patient 9 (yellow), Patient 8 (red), Patient 2 (green), patient 1 (blue) are sorted based on the initial levels of ALSFRS-R sum scores. **(A)** Courses of ALSFRS-R sum scores of the four individual patients starting at different baseline values and developed differently over the measured time span. **(B)** Long-term courses of MUNIX scores for the left and right ADM of the same four patients. Individual long-term monitoring of cortical thickness of these four patients in the precentral ventral (PreV, **C)** and dorsal cortex (PreD, **D)** and postcentral ventral (PoV, **E)** and dorsal (PoD, **F)** cortex of both hemispheres. Individual cortical thickness is *z*-transformed to age-related healthy control level (see Table 2). Lh, left hemisphere; Rh, right hemisphere.

Repeated long-term follow-up T1 MRI data exceeding 18 months were available in two leg-onset patients (patients 8, 9) and two arm-onset patients (patients 1, 2). The four patients presented different initial levels and longitudinal courses of ALSFRS-R sum scores (Figure 4A), MUNIX scores (Figure 4B), and cortical thickness alterations (Figures 4C–F). Both patients 1 and 2 have in common young-onset (21–30 years), arm-onset diagnosis, low levels of ALSFRS-R scores (patient 1: 30 score points, patient 2: 35 score points) upon first MRI scan, low MUNIX scores for left and right ADM (see Table 1), and similar progression rates (patient 1: 0.37, patient 2: 0.46). In both patients, ALSFRS-R sum scores decreased over time (patient 1: blue; patient 2: green; Figure 4A). In contrast, MUNIX for left and right ADM stagnated at low level (patient 1: blue; patient 2: green; Figure 4B). Cortical thickness of the precentral ventral cortex persisted below healthy control level over time in both patients (patient 1: blue, patient 2: green; Figure 4C). Cortical thickness of the precentral dorsal cortex decreased below healthy control level in patient 1 in the longitudinal course (Figure 4D). No cortical thinning consistently below healthy control level was found for the postcentral ROIs in both patients 1 and 2 (Figures 4E,F). Patient 8 and 9 (both aged 46–50 years) were diagnosed with leg-onset disease and obtained high ALSFRS-R sum scores at time point of initial MRI scan (Table 1). Progression rates of patient 8 (0.16) and 9 (0.00) were much lower than for patients 1 and 2. In both patients, ALSFRS-R sum scores declined over time (patient 8: red, patient 9: yellow, Figure 4A). MUNIX scores of patient 9 similarly decreased for the left and right ADM (yellow, Figure 4B). In patient 8, MUNIX scores for the left ADM started at much higher level than for the right ADM and showed a more pronounced decline of scores over time (red, Figure 4B). Patient 8 exhibited progressive cortical thinning only in the postcentral ventral cortex (Figure 4E). In patient 9, cortical thinning of the right precentral dorsal cortex spread to the left hemisphere over the time course (yellow, Figure 4D). Cortical thickness of the postcentral dorsal cortex further decreased over time (yellow, Figure 4F). Despite fluctuations, ventral sections of precentral and postcentral cortex of patient 9 persisted at healthy control level (yellow, Figures 4C,E).

## Discussion

Cortical thinning was heterogenous and most pronounced in the precentral ventral cortex. ALSFRS-R sum score was associated with both cortical thickness and MUNIX scores. Individual longitudinal monitoring of clinical ALSFRS-R, neurophysiological MUNIX, and MRI cortical thickness indicated both interindividual differences among ALS patients as well as differences in temporal dynamics between biomarkers over the course of disease.

### Cortical thickness of the precentral and postcentral cortex

Cortical thickness was highly age-dependent and significantly different between precentral and postcentral cortex as well as between ventral and dorsal subdivisions of precentral and postcentral cortex. Postmortem (37) and MRI (38, 39) studies showed approximately 1.5 times greater cortical thickness of the precentral compared to the postcentral cortex. The only study addressing gradients of postcentral cortical thickness in humans (40) reported greatest cortical thickness in the area defined as ventral segment in our study. Age effects on precentral and postcentral cortical thickness have been well described (36).

### Variability of cortical thinning

Individual cross-sectional analysis revealed heterogenous individual states of cortical thinning, which was more pronounced in the precentral than in the postcentral cortex. Postcentral cortical thinning was only present in four patients. Three out of these four patients also showed pronounced cortical thinning of the precentral cortex. These observations are consistent with studies reporting postcentral atrophy was rather less prominent or not detectable (12, 41). Instead, postcentral atrophy was discussed to result from the spread of cortical degeneration in the course of disease (41, 42). With respect to the spread of disease, interestingly, individual longitudinal analysis revealed that patient 8 developed postcentral cortical thinning despite lack of precentral cortical thinning. Cortical thinning was most pronounced in the ventral segment of the precentral cortex. The precentral ventral cortex as defined here was also reported to exhibit alterations in ALS patients in other studies (14, 43, 44). Seven out of twenty patients exhibited no indications of cortical thinning. This finding is supported by a meta-analysis reporting cortical atrophy only in a percentage of ALS cases (45) and other MRI studies failing to find alterations in the precentral cortex of ALS patients (17–19). The lack of cortical thinning in ALS patients may be explained by low progression rates and young-onset. Cortical thinning was primarily observed in ALS patients with faster progression or advanced stage of disease (46). Six out of seven patients exhibiting no indications of cortical thinning were characterized with disease progression rates less or equal to 0.40. Moreover, the ALS sample of the current study was much younger than ALS patients involved in most MRI studies (8, 13, 17, 43, 47, 48). Individual cross-sectional analysis also revealed enhanced levels of cortical thickness predominantly in the postcentral cortex. Future studies may investigate if enhanced levels of cortical thickness may be associated with processes of neuroplasticity or treatment effects. Finally, heterogeneous alterations in cortical thickness (including increases and decreases) argue for the need of individual perspective on ALS patients beyond group averages (6).

### Longitudinal monitoring of cortical thickness, ALSFRS-R, and MUNIX

Longitudinal monitoring of cortical thickness in four patients revealed differences in temporal dynamics of clinical ALSFRS-R, neurophysiological MUNIX, and MRI cortical thickness in the same individual patients. The long-term biomarker monitoring was limited to the patients who survived for longer periods of time and who underwent more than three MRI scans. All other patients of the sample received three MRI scans or less due to short survival or lack of scan capability. Similar to Abhinav et al. (49), patients showed very different baseline levels and various progression types of ALSFRS-R sum scores over time. While the decline of high-level ALSFRS-R sum scores of patient 9 (ALSFRS-R baseline: 48) was evidently observable, changes in ALSFRS-R sum scores of progressed stage patient 1 (ALSFRS-R baseline: 30) were less evident. These observations are consistent with ALSFRS-R being considered to be less sensitive for short-term time windows and slow disease progression (3, 24, 50). ALSFRS-R is also regarded as a rather general severity summary scale without sensitivity for mode of disease (17, 29). The unspecific character of ALSFRS-R may also explain why correlations between ALSFRS-R and cortical thickness were weak or not detectable (9, 15, 21). In contrast to ALSFRS-R, MUNIX significantly differentiated between arm and leg-onset of disease. Corresponding to Grimaldi et al. (26), MUNIX scores significantly correlated with ALSFRS-R scores, but showed much faster longitudinal dynamics than ALSFRS-R scores as reported by Neuwirth et al. (29). Once at very low level MUNIX scores stagnated across time in arm-onset patients 1 and 2. The phenomenon of a floor effect of MUNIX measurements at low level in completely wasted muscles was described by Neuwirth et al. (51). In contrast, higher level MUNIX scores for ADM in leg-onset patients 8 and 9 showed a fast decrease over time. Consistent with these observations, clinical markers are considered to be more sensitive to changes than MRI markers (17). Although MUNIX is considered a candidate biomarker like MUNE for LMN function (24), MUNIX scores may be influenced by both lower and UMN function (23). Existing studies investigating correlations of cortical thickness to MUNE or other neurophysiological techniques failed to find significant correlations (21, 30). As the first study combining MRI cortical thickness with MUNIX, we also found no significant correlations. Differences in both individual state of disease and within-subject temporal dynamics of various biomarkers may explain the difficulty to find significant correlations in multimodal biomarker use.

### Methodological limitations

Some methodological limitations need to be considered. First, the sample size was limited to 20 patients. As ALS MRI studies suffer from high costs and high drop-out rates due to increasing disability of patients (50), many published unicenter ALS MRI studies included samples smaller than 20 ALS patients (12, 41, 52, 53). Second, the MRI magnetic field strength was 1.5T. Although MRI magnetic field strength of 3T may have been beneficial, 1.5T still was sufficient for the detection of gray matter alterations in ALS MRI studies (11, 12, 22, 41, 54). Third, by the use of a more conservative ROI based approach than a vertex-based approach, the study may have failed to detect very focal cortical thinning inside of the ROIs. However, this approach did not only reduce the influence of false positive results but still successfully detected cortical thinning. Fourth, MUNIX scores were not assessed in leg muscles. However, this study is the first cross-sectional and longitudinal study combining cortical thickness analysis with both ALSFRS-R and MUNIX with respect to the individual patient. Fifth, longitudinal monitoring of biomarkers was limited to four patients of the sample due to high disability (*n* = 6), death (*n* = 4), lack of T1 data (*n* = 6). Still, our long-term biomarker monitoring analysis is unique, as to our knowledge, none of the published longitudinal MRI studies showed longitudinal courses of both MRI cortical thickness and MUNIX biomarkers using as many repeated measures in a time course longer than 18 months as presented in the current study. Moreover, most MRI studies focused on group analysis irrespective of the individual patient (9, 10, 14–17), although the individual perspective has been increasingly demanded in ALS neuroimaging research (6, 24, 55, 56).

## Conclusions

The present study demonstrated that MRI is a potential biomarker for the differentiation of individual states of cortical thinning in an ALS sample including young-onset and slow progressive patients. Longitudinal monitoring of MRI, clinical, and neurophysiological biomarkers in the same patient reveal substantial differences in temporal dynamics. Combinatory biomarker use contributes a substantial gain of information about individual state of disease beyond group averages. Future studies may expand the idea of combining neuroimaging techniques with other clinical or molecular biomarkers to deepen our understanding of multisystem/multifactorial ALS disease progression.

## Author contributions

AW: substantial contribution to the conception and the design of the study, acquisition, analysis, interpretation of the MRI data, and composition of the manuscript. AK and DB: substantial contribution to the conception of the study, data acquisition, interpretation of the data, and revision of the manuscript. IK, TG, SJ, WS-M, T-HB, and UB: substantial contribution to the conception of the study, acquisition, analysis, and interpretation of clinical and neurophysiological data, and revision of the manuscript. SJ, OH, AK, DB, WS-M, and UB: care for ALS patients in outpatient clinic, treatment concept. MG: substantial contribution to the conception of the study, supervision of MRI data analysis and data interpretation, and revision of the manuscript.

### Conflict of interest statement

The authors declare that the research was conducted in the absence of any commercial or financial relationships that could be construed as a potential conflict of interest.

## Abbreviations

ADM: abductor digiti minimi
ALS: amyotrophic lateral sclerosis
ALSFRS-R: ALS-specific functional rating scale revised
CMAP: compound muscle action potential
G-CSF: granulocyte-colony stimulating factor
LMN: lower motor neuron
MNI: Montreal Neurological Institute
MPRAGE: magnetization prepared rapid gradient echo sequence
MRI: magnetic resonance imaging
MUNE: motor unit number estimation
MUNIX: motor unit number index
PoD: postcentral dorsal
PoV: postcentral ventral
PreD: precentral dorsal
PreV: precentral ventral
ROI: region of interest
SIP: surface interference pattern
SMN: sensorimotor network
SMUP: single motor unit potential
UMN: upper motor neuron.
